# Value of nitroglycerin test in the diagnosis of heart failure in emergency department patients with undifferentiated dyspnea

**DOI:** 10.1002/clc.23615

**Published:** 2021-06-02

**Authors:** Adel Sekma, Khaoula Bel Haj Ali, Camilia Jeddi, Nadia Ben Brahim, Nasri Bzeouich, Imen Gannoun, Imen Trabelssi, Kamel Laouiti, Mohamed Habib Grissa, Kaouthar Beltaief, Dridi Zohra, Zorgati Asma, Boukadida Lotfi, Youssef Rym, Houda Ben Soltane, Mezgar Zied, Khrouf Mariem, Mohamed Amine Msolli, Boukef Riadh, Wahid Bouida, Hamdi Boubaker, Semir Nouira

**Affiliations:** ^1^ Emergency Department Fattouma Bourguiba University Hospital Monastir Tunisia; ^2^ Research Laboratory LR12SP18 University of Monastir Monastir Tunisia; ^3^ Cardiology Department Fattouma Bourguiba University Hospital Monastir Tunisia; ^4^ Emergency Department Sahloul University Hospital Sousse Tunisia; ^5^ Emergency Department Farhat Hached University Hospital Sousse Tunisia

**Keywords:** diagnosis, dyspnea, heart failure, nitroglycerin

## Abstract

**Background:**

Rapid diagnosis of heart failure (HF) in acutely dyspneic patients can be challenging for emergency department (ED) physicians.

**Hypothesis:**

Cardiac output (CO) change with sublingual nitroglycerin (NTG) could be helpful in the diagnosis of HF in patients with acute undifferentiated dyspnea.

**Materials and Methods:**

A prospective study of patients >18 years admitted to the ED for acute dyspnea. Using thoracic bioimpedance, we measured CO change at baseline and after sublingual administration of 0.6 mg of NTG. HF was defined on the basis of clinical examination, pro‐brain natriuretic peptide levels, and echocardiographic findings. Diagnostic performance of delta CO was calculated by sensitivity, specificity, likelihood ratio and receiver operating characteristic (ROC) curve.

**Results:**

This study included 184 patients with mean age of 64 years. Baseline CO was comparable between the HF group and the non‐HF group. At its best cutoff (29%), delta CO showed good accuracy in the diagnosis of HF with a sensitivity, specificity, positive and negative likelihood ratios of 80%, 44%, 57%, and 66% respectively. Area under ROC curve was 0.701 [95% CI 0.636–0.760]. The decrease of CO with sublingual NTG was significantly higher in patients with HFpEF compared with those with HFrEF. Multivariate analysis, showed that delta CO was an independent factor associated with HF diagnosis [OR 0.19 (95% CI 0.11–0.29); p < .001].

**Conclusions:**

Our study showed that CO change with sublingual nitroglycerin is a simple tool that may be helpful for the diagnosis of HF in ED patients with undifferentiated dyspnea.

## 1 INTRODUCTION

Heart failure (HF) is among the most costly chronic diseases with a major impact on the patients' quality of life. Its evolution is characterized by recurrent exacerbations which represent a frequent reason for admission to the emergency department (ED).^1^ Dyspnea is the main symptom of HF exacerbations, but differentiation of dyspnea related to HF from other causes can be challenging despite improved diagnostic tools ranging from chest X‐ray to more sophisticated and less accessible techniques such as cardiac ultrasound or pulmonary arterial catheterization.^2^ Early diagnosis is nevertheless a prerequisite for appropriate management. Delays in establishing the correct diagnosis and appropriate treatment may influence subsequent length of hospital stay, morbidity, and mortality.^3^, ^4^ Numerous studies have shown that the measurement of cardiac output (CO) or its surrogate after preload‐ modifying manoeuvers could be a reliable and accurate method to investigate hemodynamic status. Depending on cardiac function, a given change in preload could lead to either a significant or a negligible increase in stroke volume.^5^, ^6^, ^7^, ^8^ In a normal heart, the more the left ventricle is filled, the better it contracts and the higher is the volume of systolic ejection (steep slope of the Frank‐Starling curve). Preload dependence of CO is less detectable in the failing heart with regard to the flat cardiac function curve.^9^, ^10^, ^11^ Postural maneuvers that change cardiac venous return have been proposed as a useful means for the diagnosis of HF in dyspneic patients, but this has rarely been described with pharmacological interventions.^12^, ^13^, ^14^ The aim of our study was to evaluate the utility of sublingual nitroglycerin administration (NTG test) and its effects on non‐invasive cardiac output measurement in the diagnosis of acute HF in ED patients presenting with acute dyspnea.

## 2 PATIENTS AND METHODS

### 2.1 Design of the study

This is a prospective descriptive study conducted in the Emergency Department of Fattouma Bourguiba University Hospital in Monastir from January 2019 to February 2020. Patients over the age of 18 years presenting to the ED with dyspnea were included. Major exclusion criteria were as follows: acute myocardial infarction or ischemic chest pain within the prior 24 h, pericardial effusion, chest wall deformity, coma, need for mechanical ventilation or vasopressor drugs, serious and sustained arrhythmia, pace maker, severe mitral valve disease, severe pulmonary arterial hypertension, renal failure (creatinine >350 μmoL/L). The study was approved by the ethics committee of our institution; informed consent was obtained before the start of the protocol in all included patients. The study was registered in the ClinicaTrials.gov register under the number NCT04484298.

### 2.2 Participants and methods

Patients' demographic characteristics as well as clinical examination data including age, sex, height, weight, history, current drug treatment, blood pressure, heart rate, respiratory rate, SpO2, and temperature were collected. Standard biological examinations and blood gases as well as cardiac enzymes and the pro‐brain natriuretic peptide (pro‐BNP) were measured at admission. The definitive diagnosis of HF was made by two clinical experts on the basis of clinical data, echocardiographic findings and pro‐BNP level. CO measurement was performed by transthoracic impedance using the Biopac system (Biopac Student Lab software version 3.7.2). Artifacts in the bioimpedance signal were detected and excluded from the study. In practice, the device was connected using four electrodes placed at the base of the neck (posterior) and at the base of the thorax (posterior). At each level, two electrodes were placed 5 cm apart and the 2 levels were separated by a distance of 28 cm. The electrocardiographic recording was taken simultaneously with 2 other electrodes placed at the level of the right upper limb and lower left limb. The parameter measured by the transthoracic impedance was stroke volume (SV). It was calculated from the formula: SV = VTEP × VET × ([dZ/dtmax]/ BCI) where VTEP is the volume of participating electrical tissue, VET is the left ventricular ejection time, dZ/dtmax is the rate of impedance change during systole, and BCI is the baseline chest impedance. Cardiac output was calculated instantaneously from the systolic ejection volume (SEV) and the cardiac frequency. The device was set up in such a way that data acquisition was averaged over 10 cardiac cycles. The average of 3 measurements of the SEV was retained provided that the values did not differ more than 10% from each other. All the measurements were made by the same investigators who were not aware of the clinical and biological details of the patient. The treating physicians were blinded to the results of the thoracic bioimpedance. Each patient was initially placed in a semi‐sitting position at 30° for 5 min and then CO is measured (baseline CO). NTG (0.6 mg) was then given to the patient sublingually and CO measurement was repeated. CO was calculated by averaging three measurements at 1 min intervals at baseline and after NTG administration. Delta CO was defined as the percent of change of baseline CO after NTG test.

#### 2.2.1 Statistical analysis

Variables were expressed as mean ± standard deviation, or median and interquartile range as appropriate. The patients were divided into two groups: a group of patients identified as having HF (HF group) and a group of patients where the diagnosis of HF was excluded (non HF group). Comparisons between both groups were made among continuous variables using Student's *t* test for independent samples. Chi‐square was used for discrete variables. Receiver operating characteristic (ROC) curves for predicting HF were constructed and the area under the curve (AUC) was measured for delta CO. Sensitivity, specificity, positive and negative predictive values, and likelihood ratios of positive and negative results were calculated using the optimal cutoff value of delta CO. Multivariate logistic regression analysis was used to identify the independent predictors of HF in patients with acute dyspnea. Age, sex, and clinical variables that were statistically significant after univariate analysis were included in this analysis. Each variable of interest was examined with univariate analysis, and variables that were significant at the 0.20 level were included in the logistic regression. We estimated a sample size of 110 subjects with acute dyspnea, assuming an anticipated delta CO sensitivity of at least 90% with a power (β) = 80%, and a probability of type‐1 error (α) = 0.05. A p‐value of .05 was considered statistically significant. Calculations were performed with SPSS version 20 software package for Windows (SPSS Inc, Chicago, IL).

## 3 RESULTS

**FIGURE 1:**
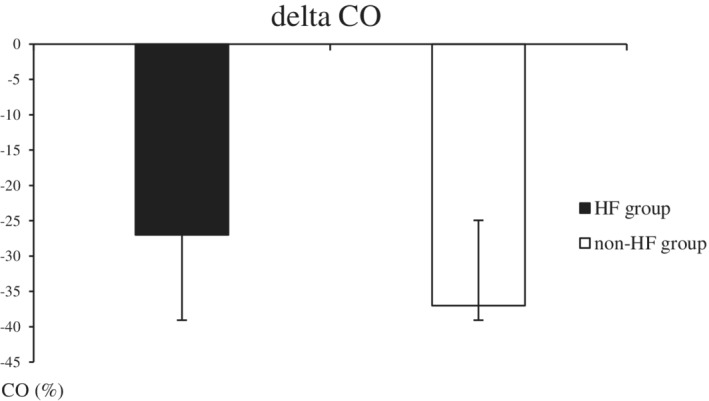
Comparison of cardiac output change in heart failure and non‐heart failure patients between baseline and after nitroglycerin test

**FIGURE 2:**
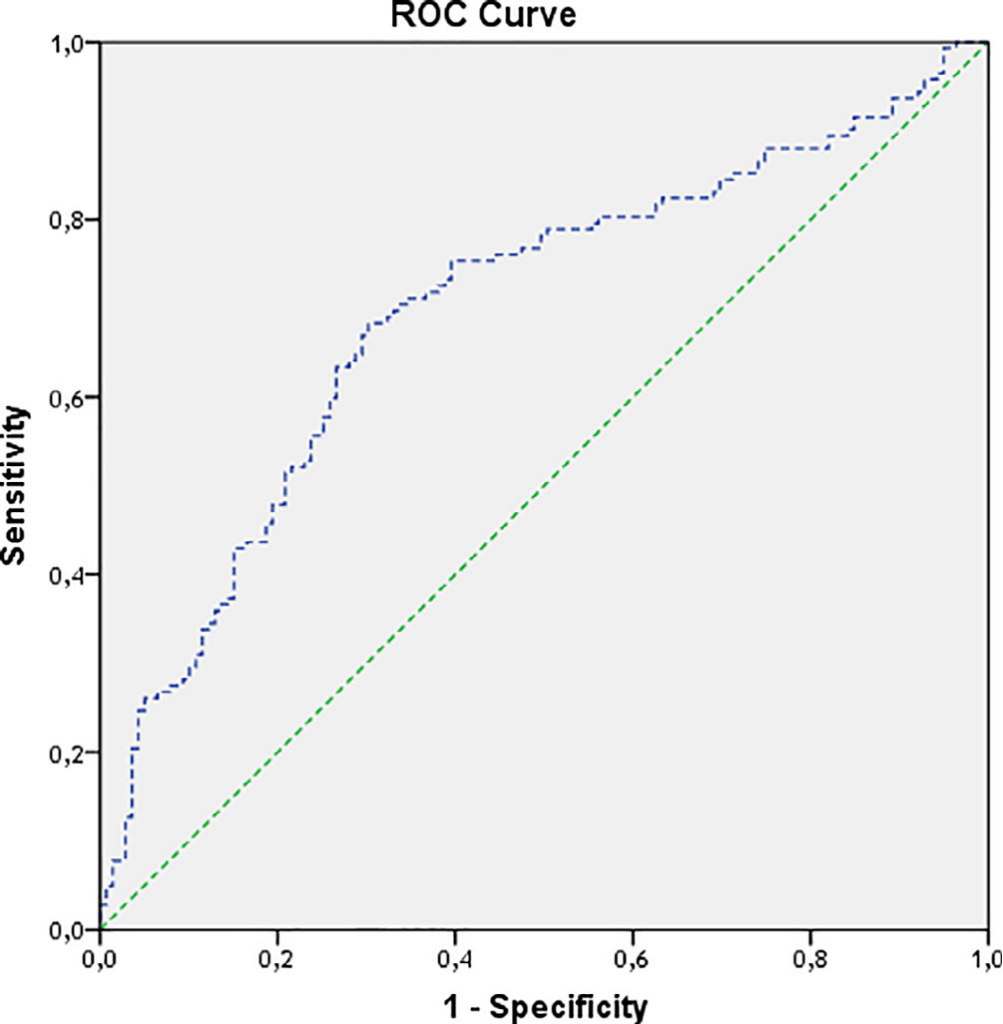
Receiver operating characteristic curve of delta cardiac output (cardiac output difference at baseline and after nitroglycerin test) for the diagnosis of heart failure

Two hundred and eighty‐four patients were included Table 1 summarizes the demographic and clinical characteristics of the overall population and the two study groups. Patients in the HF group (*n* = 143) were significantly older, with significantly higher rates of diabetes, hypertension, heart failure and coronary disease. Male predominance was observed in both groups. Mean left ventricular ejection fraction was 48 ± 14% and 63 ± 12% in the HF and the non‐HF groups respectively. In the HF group, 56 (39%) had reduced LVEF (HFrEF), and 86 (61%) had preserved LVEF (HFpEF). Baseline CO was comparable between the two groups (6.08 and 6.04 L/min respectively for the HF group and the non‐HF group. Cardiac output decreased significantly in both groups, but this decrease was greater in the non‐HF group (Figure 1). In patients with HF, baseline CO was not significantly different between the HFrEF and HFpEF groups (6.0 ± 1.3 versus 5.9 ± 1.1, respectively). Cardiac output decreased significantly in both sub‐groups but the decrease was significantly higher in the HFpEF group compared with the HFrEF group (−45 ± 1.5 versus −25.3 ± 1.3 respectively; p < .001) (Figure 1). The value of delta CO in predicting HF had an area under the ROC curve of 0.701 [95% CI 0.636–0.760] (Figure 2). Using the best cutoff value of delta CO (29%), the sensitivity, specificity, positive and negative predictive values were respectively 80%, 44%, 57%, and 66%. The results of multivariate analysis are shown in Table 2. It was found that a delta CO >29% was associated with a decrease in the probability of HF [OR 0.19 (95% CI 0.11–0.29); p < .001]. Previous history of chronic heart failure, hypertension, and coronary artery disease were the other independent factors associated with HF diagnosis on multivariate analysis.

**TABLE 1:** Patients' baseline characteristics

	Heart failure *n* = 143	Non‐heart failure *n* = 141	p
Age, years ± SD	65.2 ± 12.3	61.1 ± 14.7	.007
Gender F/M	65/78	44/97	.014
Body mass index (kg/m^2^): mean ± SD	28 ± 0 .03	25 ± 0.02	0.199
Comorbidities, *n* (%)			
Chronic heart failure	30 (21.2)	10(7.1)	.001
Coronary artery disease	34 (24.1)	9 (6.4)	<.001
Hypertension	96 (67.1)	50 (35.5)	<.001
Diabetes	71 (49.6)	43 (30.1)	.001
Atrial fibrillation	25 (17.6)	14 (10.3)	.079
Clinical parameters, mean ± SD			
SBP (mmHg)	146 ± 40	140 ± 35	0.13
DBP (mmHg)	80 ± 25	77 ± 21	0.380
Heart rate, bpm	99 ± 2	106 ± 24	.023
Respiratory rate, cpm	26 ± 7.5	26 ± 6.3	0.766
Pulse oxygen saturation, %	89 ± 10	87 ± 11	0.218
Biology, mean ± SD			
Serum sodium (mmol/L)	136 ± 4	137 ± 4	.032
Serum potassium (mmol/L)	4.4 ± 0.8	4.2 ± 0.8	.033
Glycemia (mmol/L)	11.3 ± 6.1	9.8 ± 5.4	.086
Hemoglobin (g/dl)	13.8 ± 1.6	12.8 ± 1.9	0.399
Nt proBrain natriuretic peptide (ng/L): median (IQR)	2134 (206–4460)	400 (123–1180)	<.001
LVEF (%): mean ± SD	47.9 ± 13.8	63.2 ± 12.2	<.001

Abbreviations: F/M: female/male, SD: standard deviation,SBP: Systolic Blood Pressure, DBP: Diastolic Blood Pressure, LVEF: Left Ventricular Ejection Fraction.

**TABLE 2:** Factors associated with the diagnosis of heart failure: Multivariate analysis

	Odds ratio	95% confidence interval	p
Age	1.02	0.99–1.04	.88
Sex	0.97	0.88–1.10	0.14
Chronic heart failure	3.41	1.6–7.4	.001
Hypertension	3.77	2.27–6.07	<.001
Diabetes	2.27	1.40–3 .70	.001
Coronary artery disease	4.62	2.12–10.06	<.001
Atrial fibrillation	1 .86	0 .92–3.75	.08
Serum sodium	0.94	0.89–1.01	.09
Hemoglobin	1.01	0.96–1.06	0.39
Delta CO	0.19	0.11–0.29	<.001

Abbreviation: CO, cardiac output.

## 4 DISCUSSION

Our study showed that patients with HF exhibited a lower decrease in CO after NTG test compared with the non‐HF patients consulting in ED for acute dyspnea.

Heart failure diagnosis in patients consulting for undifferentiated dyspnea is frequently challenging in the ED.^15^ In fact, available data showed that clinical history, symptoms, physical examination, chest radiography, and electrocardiography, on their own, lack discriminatory value in excluding or establishing the diagnosis of AHF in ED patients.^2^, ^16^ Certainly, brain natriuretic peptide (BNP) testing is now a routine component of the diagnostic workup of patients with possible AHF. However, even when BNP testing is incorporated into the clinical approach of acute dyspnea, misclassification remains possible. BNP is neither sensitive nor specific enough to make this marker the only way to diagnose HF. Although BNP has an established ''rule‐out'' value, elevated levels of BNP do not automatically confirm the diagnosis of AHF as elevated levels of BNP may be associated with a wide variety of cardiac and non‐cardiac causes. Besides, it is well known that there is a range of BNP values that is considered to be a 'gray zone' and a BNP value included in this range is difficult to interpret. It has been suggested that cardiac failure can be detected by measuring CO changes under pharmacological interventions that can modify the loading condition. In patients with normal cardiac function, preload changes induce significant variations of SV. As these patients are on the ascending part of the Startling curve, their CO would decrease in response to preload reduction induced by NTG.^17^, ^18^ Conversely, the same change of preload would lead to a small alteration in SV in patients with HF because they are at or near to the plateau of the heart's Starling curve.^19^, ^20^ This difference explains the ability of noninvasive measurements of CO and their change in response to NTG test to diagnose acute decompensated heart failure from other causes of acute dyspnea in patients in the ED. In our study, CO decreased significantly after NTG test in non‐HF groups, but it did not change significantly in HF patients. Traditionally, venodilation and venous pooling of blood into the lower extremities and splanchnic vessels with a decrease in CO, have been regarded as major hemodynamic effects of NTG specifically at low doses.^21^, ^22^, ^23^ However, NTG administration would not always induce a decrease in CO as reflex tachycardia and vasoconstriction could modulate and even counteract the preload effect of NTG. In our study, more than 60% of patients without HF demonstrated a very small change of CO.^24^ At higher doses, NTG is associated with dilation effect of the systemic arteries and arterioles, and this effect would increase CO.^25^ The status of left ventricular function is another important factor determining the overall hemodynamic response to NTG. In the presence of HF, CO changes are moderate because the arterial dilating properties of NTG would minimize its preload reduction effect. In addition, in patients with impaired ventricular function, sympathetic discharge is usually moderate after nitrate administration.^26^ Interestingly, NTG test seems to be more adapted to the diagnosis of HFpEF. In fact, NTG administration provoked a steeper fall in CO in patients with preserved LVEF compared to HF patients with reduced LVEF. This would be expected if we assumed that reduced cardiac compliance and impaired diastolic relaxation make the heart more dependent on cardiac preload to adequately fill the left ventricle.^27^, ^28^ The distinction between HFpEF and HFrEF is an important issue with regard to the potential specific management and clinical outcomes of these two HF phenotypes. Diagnosing HFpEF without echocardiography facilities is often challenging and the best diagnostic approach remains unclear. However, echocardiography is not always easily accessible in the ED, and measurements depend on the operator experience. Nonetheless, Both HFpEF and HFrEF are diverse disorders with variable pathophysiology. With the numbers of patients studied here and the absence of invasive hemodynamic data, the question of its applicability remains uncertain.

Our study has some limitations that deserve to be noted. Firstly, not all dyspneic patients were included, mainly those with a severe condition and those for whom CO measurement was not possible. Thus, our results may only be applicable to patients with less severe dyspnea. Of note, measurement of CO by transthoracic impedance has its own limitations that must be considered. Measurement errors could be observed in conditions such as obesity, when the location of the electrodes is not appropriate, and when the patients move during the measurement procedure. We tried to avoid these potential artifacts as much as possible. Nevertheless, the accuracy of this measurement system and its agreement with other techniques has been well demonstrated.^29^, ^30^ Moreover, to overcome the problem of CO absolute values accuracy in the present study, we expressed our results by the percentage of CO change from baseline. Secondly, in our study, CO measurement was performed while treatment was ongoing. Therefore, we cannot exclude the fact that our results could have been influenced by the initial treatment.

## 5 CONCLUSION

In this study, we showed that NTG test coupled to a fast and non‐invasive measurement of CO may be a significant aid to the conventional diagnostic approach of HF. However, further studies are needed to validate our findings.

## CONFLICT OF INTEREST

The authors declare no potential conflict of interest.

## AUTHOR CONTRIBUTION

Msolli Mohamed Amine, Boubaker Hamdi, and Sekma Adel performed statistical analysis; N.F, Kaouthar Beltaief, Bzeouich Nasri, Gannoun Imen, Trabelssi Imen, Laouiti Kamel, and MH.G supervised the study; Msolli Mohamed Amine, Kaouthar Beltaief, A.B acquired the data; Nouira Semir, Z.D, conceived and designed the research; S.N drafted the manuscript; M.M, Boukef Riadh and Bouida Wahid made critical revision of the manuscript for key intellectual content.

## Data Availability

Research data are not shared.
